# Non-peptidergic small diameter primary afferents expressing VGluT2 project to lamina I of mouse spinal dorsal horn

**DOI:** 10.1186/1744-8069-7-95

**Published:** 2011-12-08

**Authors:** Jennifer N Clarke, Rebecca L Anderson, Rainer V Haberberger, Ian L Gibbins

**Affiliations:** 1 Anatomy and Histology, and Centre for Neuroscience, Flinders University, GPO Box 2100, Adelaide, SA, 5001, Australia

**Keywords:** nociceptors, primary afferent neurons, VGluT2, CGRP, microdomain, spinal dorsal horn

## Abstract

**Background:**

Unmyelinated primary afferent nociceptors are commonly classified into two main functional types: those expressing neuropeptides, and non-peptidergic fibers that bind the lectin IB4. However, many small diameter primary afferent neurons neither contain any known neuropeptides nor bind IB4. Most express high levels of vesicular glutamate transporter 2 (VGluT2) and are assumed to be glutamatergic nociceptors but their terminations within the spinal cord are unknown. We used *in vitro *anterograde axonal tracing with Neurobiotin to identify the central projections of these putative glutamatergic nociceptors. We also quantitatively characterised the spatial arrangement of these terminals with respect to those that expressed the neuropeptide, calcitonin gene-related peptide (CGRP).

**Results:**

Neurobiotin-labeled VGluT2-immunoreactive (IR) terminals were restricted to lamina I, with a medial-to-lateral distribution similar to CGRP-IR terminals. Most VGluT2-IR terminals in lateral lamina I were not labeled by Neurobiotin implying that they arose mainly from central neurons. 38 ± 4% of Neurobiotin-labeled VGluT2-IR terminals contained CGRP-IR. Conversely, only 17 ± 4% of Neurobiotin-labeled CGRP-IR terminals expressed detectable VGluT2-IR. Neurobiotin-labeled VGluT2-IR or CGRP-IR terminals often aggregated into small clusters or microdomains partially surrounding intrinsic lamina I neurons.

**Conclusions:**

The central terminals of primary afferents which express high levels of VGluT2-IR but not CGRP-IR terminate mainly in lamina I. The spatial arrangement of VGluT2-IR and CGRP-IR terminals suggest that lamina I neurons receive convergent inputs from presumptive nociceptors that are primarily glutamatergic or peptidergic. This reveals a previously unrecognized level of organization in lamina I consistent with the presence of multiple nociceptive processing pathways.

## Background

Most primary afferent neurons in dorsal root ganglia (DRG) are considered to use glutamate as their fast excitatory transmitter, [1-3], although some may use aspartate instead [4-6]. Many smaller DRG neurons, including nociceptors, express high levels of neuropeptides, usually both calcitonin gene-related peptide (CGRP) and substance P [7-12] or bind the lectin, IB4 [13-15]. The peptidergic neurons span a range of nociceptive modalities, including those associated with transient receptor potential cation channel subfamily V (TRPV1) receptor activation in response to noxious heat or inflammation, for example [16,17].

Early microscopic identification of putative glutamatergic primary afferent neurons attempted to directly localize glutamate or glutaminase, for example [2,6,18-20], which also exist in metabolic pools not directly associated with neurotransmission. The subsequent discovery of the vesicular glutamate transporters (VGluTs) enabled specific immunolabeling of presumptive glutamatergic neurons based on their expression of VGluTs [21-24]. Thus, VGluT1 is expressed mainly by large diameter primary afferent neurons, mostly representing myelinated mechanoceptors, whereas small diameter primary afferent neurons, including unmyelinated nociceptors, express VGluT2 to varying degrees [23,25,26].

VGluT2 is considered to be expressed at low levels in most peptidergic nociceptors [24,27-31]. However, there is a significant population of small diameter neurons, presumably nociceptors, that express high levels of VGluT2 in their soma but no known neuropeptide nor bind IB4 [30]. It has proven difficult to positively identify the central terminations of these VGluT2-immunoreactive (IR) nociceptors mainly because (1) the level of immunolabeling in their terminals may be low; and (2) these terminals are greatly outnumbered by intrinsic spinal terminals that express strong VGluT2-IR [24,26-28,30]. Therefore, we developed an *in vitro *technique for anterograde axonal tracing of dorsal roots using Neurobiotin which labels unmyelinated as well as myelinated sensory fibers entering the dorsal horn. By combining Neurobiotin tracing of dorsal roots with immunohistochemical localization of VGluT2, we have distinguished the central projections of small diameter glutamatergic primary afferents from intrinsic VGluT2-IR endings in mouse lumbar spinal dorsal horn. Using three-dimensional (3D) quantitative confocal microscopy, we have compared the organization of central terminals of the subset of VGluT2-IR afferents which lack CGRP-IR (and IB4-binding) with those of afferents containing CGRP-IR. Our data show that a discrete population of putative non-peptidergic glutamatergic nociceptors terminate in lamina I that can be distinguished from afferent terminals expressing neuropeptides. Furthermore, lamina I was found to consist of a series of microdomains that are enriched in terminals which are predominantly either peptidergic or presumptive glutamatergic nociceptors.

## Results

### Distribution of VGluT2- and CGRP-immunoreactive terminals in the dorsal horn

**Figure 1:**
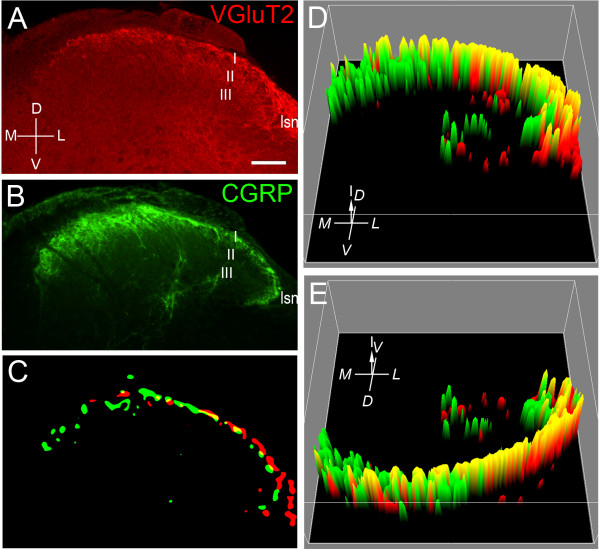
**VGluT2 and CGRP immunoreactivity in the dorsal horn of L3 spinal cord**. ***A: ***VGluT2-IR labeling occurs throughout the gray matter but labels a dense band of terminals in lamina I which is more prominent laterally. VGluT2-IR is also prominent in the lateral spinal nucleus and lamina II. ***B: ***Strong CGRP-IR occurs across lamina I but is especially dense medially. Some CGRP-IR fibers project deeper within the dorsal horn around the mid regions of laminae III-IV. ***C: ***Images in (***A***) and (***B***) band-pass filtered by Fast Fourier Transformation (FFT), thresholded and merged to reveal larger scale domain organization of the VGluT2-IR (red) and CGRP-IR (green) terminals in lamina I. Domains enriched with VGluT2-IR or CGRP-IR terminals are interspersed with domains containing both types of terminals (yellow). VGluT2-IR domains are superficial to CGRP-IR domains in lateral dorsal horn. ***D-E: ***Intensity plots of merged and aligned FFT-filtered images of L3 spinal cord from three animals to show the overall organization of domains enriched for VGluT2-IR or CGRP-IR. The height of the peak represents the mean labeling intensity of the domain; red, VGluT-IR; green, CGRP-IR. ***D: ***Ventral view. ***E: ***Dorsal view. Domains enriched in VGluT2-IR tend to lie more dorsally, especially in lateral lamina I where it abuts the lateral spinal nucleus. Domains enriched in CGRP-IR tend to lie more deeply, especially in medial lamina I. Scale bar in ***A ***represents 100 μm and applies to all panels. The axes indicate the orientation of the transverse sections: D; dorsal, V; ventral, M; medial, L; lateral, and for the 3D plots, I; intensity in arbitrary grayscale units. The approximate location of the superficial laminae are marked in ***A ***and ***B***: lamina I; I, lamina II; II, and lamina III; III. The location of the lateral spinal nucleus is also indicated; lsn.

Wide-field multiple-labeling immunofluorescence at lumbar segments of mouse spinal cord showed that VGluT2-IR varicosities were abundant throughout the spinal gray matter (Figure 1A), consistent with prior observations [24,25,27-30,32]. The VGluT2 immunolabeling clearly distinguished spinal regions containing terminals with different labeling intensities. As has been previously reported, intensely labeled VGluT2-IR terminals were particularly dense in lamina I and in the lateral spinal nucleus, with a dense band of VGluT2 terminals also evident in lamina II [28-30]. Within lamina I, VGluT2-IR varicosities were most abundant laterally, where they formed a band extending deeper into the dorsal horn compared with medial lamina I. As reported previously [7,28,30] CGRP-IR varicosities were prominent across lamina I and in the central region of lamina IV-V (Figure 1B).

High-magnification examination of lamina I sections double-labeled for VGluT2-IR and CGRP-IR suggested that clusters of VGluT2-IR varicosities and CGRP-IR varicosities often occupied small mutually exclusive regions or domains. Fourier transformation and spatial filtering of images to identify large-scale features of the VGluT2-IR and CGRP-IR labeling in lamina I confirmed that domains enriched for VGluT2-IR were interspersed with domains enriched for CGRP-IR, with limited overlap, mostly in lateral domains (Figure 1C). Observation of consecutive transverse sections showed rostral-caudal variation in the precise medio-lateral location of the VGluT2-IR-rich and CGRP-IR-rich domains. Therefore in order to demonstrate the overall organisation of these domains, Fourier transformed images from 3 animals were aligned and merged (Figure 1D-E). The resultant composite image also showed that VGluT2-IR-enriched domains tended to be superficial to CGRP-IR-enriched domains, especially in lateral lamina I (Figure 1C-E). An additional analysis of Fourier transformed images from consecutive sections from one animal confirmed that domains enriched in VGluT2-IR were more prominent in the lateral regions of lamina I, compared with CGRP-IR-enriched domains (two-way ANOVA, significant interaction between label and region: F_(1,159) _= 6.1, p = 0.02; n = 163 domains from five sections taken from lumbar spinal segment L3). There was also a significant positive correlation between the medial-to-lateral location of the VGluT2-IR domains and their size (r = 0.4, p < 0.001; n = 102 domains from five sections at L3).

### Anterograde axonal tracing of primary afferent fibers

To distinguish VGluT2-IR terminals of primary afferent origin in the dorsal horn from those of intrinsic origin, VGluT2 immunolabeling was combined with anterograde axonal tracing from the dorsal roots with Neurobiotin. Neurobiotin always labeled fibers projecting into the dorsal horn and dorsal funiculus. The following observations are taken from 6 preparations showing uniformly extensive labeling with Neurobiotin as well as strong immunoreactivity for VGluT2 and CGRP.

**Figure 2:**
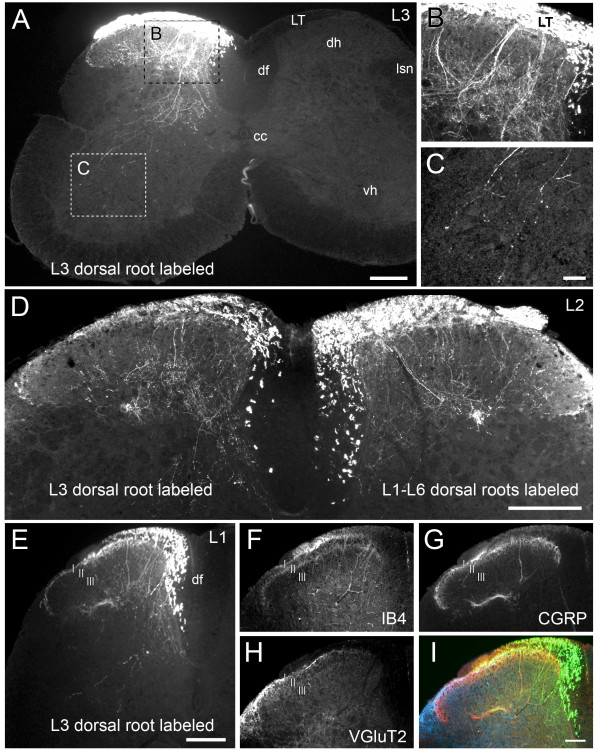
**Central terminals of primary afferent fibers detected by anterograde axonal tracing with Neurobiotin**. ***A: ***Neurobiotin labeling in L3 spinal cord after application of the tracer to L3 dorsal root. There is extensive Neurobiotin labeling of axons in Lissauer's Tract, large and fine-diameter varicose fibers in the dorsal horn (boxed region enlarged in ***B***) and extending into the ventral horn (boxed region enlarged in ***C***). ***D: ***Neurobiotin labeling in L2 dorsal horns after application of the tracer to L3 dorsal root on one side of the spinal cord and all six lumbar dorsal roots on the other side. Tracing from multiple roots produces more labeled fibers in the dorsal horn but with a comparable distribution compared with labeling from a single root. ***E: ***Neurobiotin labeling in L1 dorsal horn after application of the tracer to the ipsilateral L3 dorsal root. There is strong labeling in the dorsal funiculus, but labeling can still be seen in lamina I. The same section is labeled for IB4-binding (***F***), CGRP-IR (***G***), VGluT2-IR (***H***); overlaid image (***I***; Neurobiotin; green, IB4; orange, CGRP; red, VGluT2; blue). Representative images from 3 separate experiments; ***A-C***, ***D***, and ***E-I***. Scale bar represents 200 μm in ***A***, ***D ***and ***E***, 20 μm in ***C ***(also applies to ***B***), and 100 μm in ***I ***(also applies to ***F-H***). Lissauer's Tract; LT, dorsal horn; dh, ventral horn; vh, dorsal funiculus; df, central canal; cc, lateral spinal nucleus; lsn, lamina I; I, lamina II; II, lamina III; I.

Neurobiotin labeled large diameter fibers (presumptive myelinated mechanoreceptors) and fine varicose fibers (presumptive Aδ- and C-fibers) throughout the ipsilateral dorsal horn. Fine varicose fibers labeled with Neurobiotin were particularly dense in the superficial dorsal horn. A plexus of large and small diameter Neurobiotin-labeled fibers ramified in the deep dorsal horn corresponding to laminae III-IV. Some labeled fibers extended into the ventral horn. Occasional labeled fibers followed the border of the dorsal funiculus (Figure 2A-C).

In the superficial dorsal horn, Neurobiotin-labeled primary afferent fibers extended at least three spinal segments cranial and at least two spinal segments caudal to the dorsal root where tracer was applied, as expected from previous observations using other tracing methods [33-35]. Neurobiotin labeling of fibers in the deep dorsal horn and the ventral horn was most extensive at and immediately caudal to the level of the traced dorsal root. In all experiments, subsets of Neurobiotin-labeled primary afferent terminals also were labeled for VGluT2-IR, CGRP-IR or IB4-binding (Figure 2E-I). As expected from previous studies [13-15,25,30], IB4-binding fibers terminated deeper within the dorsal horn than the CGRP-IR fibers; they were not investigated further here. Neurobiotin labeling was not observed in the lateral spinal nucleus in any experiment, consistent with earlier lesioning studies that concluded there is no direct primary afferent projection to this area [36].

Simultaneous application of Neurobiotin to all six lumbar dorsal roots (L1-L6) labeled more primary afferent terminals compared with Neurobiotin application to only the L3 dorsal root (simultaneous L1-L6 application: Neurobiotin labeled 52 ± 8% of CGRP-IR terminals at L3; n = 3; only L3 application: Neurobiotin labeled 36 ± 4% of CGRP-IR terminals at L3; n = 3). However, the relative distributions of total Neurobiotin-labeled fibers, Neurobiotin-labeled CGRP-IR terminals and Neurobiotin-labeled VGluT2-IR terminals across the superficial dorsal horn did not differ between experiments, thereby permitting combined analysis of all six of these experiments.

### VGluT2-IR primary afferent fibers terminate predominantly in outer lamina I

High resolution confocal microscopy showed that the band of VGluT2-IR terminals in upper lamina I included a range of terminal sizes and labeling intensities, with many intensely VGluT2-IR labeled varicosities. The second band of VGluT2-IR varicosities in outer lamina IIcomprisedVGluT2-IR varicosities which were overall less dense and less intensely labeled than those in upper lamina I. Consistent with low magnification studies by us (Figure 2) and others [28-30], VGluT2-IR varicosities were also distributed at lower density throughout the remaining spinal gray matter, including inner lamina I and inner lamina II.

**Figure 3:**
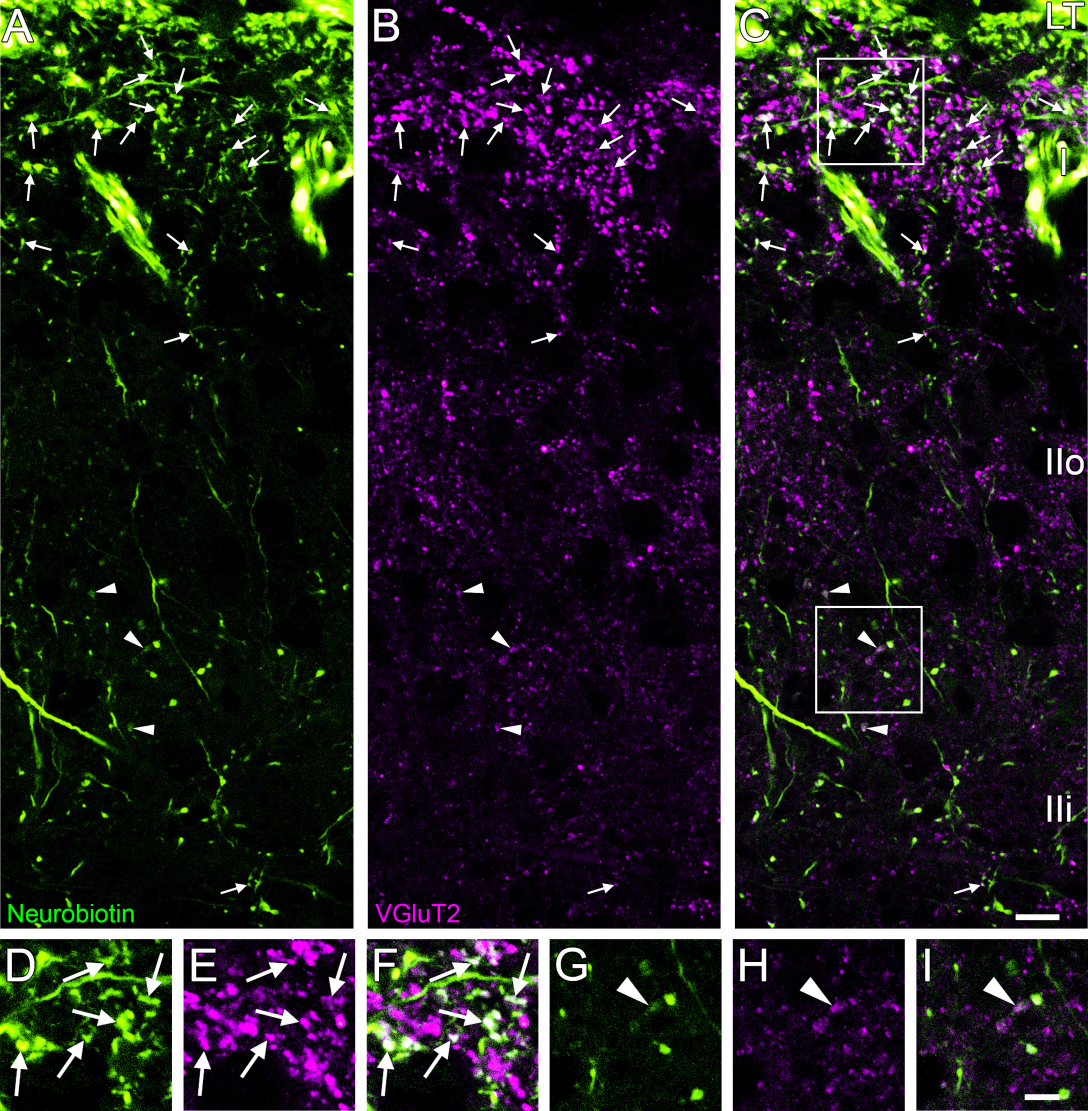
**Identification of the central terminals of VGluT2-expressing primary afferents**. High resolution confocal microscope imaging was performed to identify VGluT2-IR terminals labeled with Neurobiotin and therefore of primary afferent origin. Images are a projection of 3 optical z-sections captured 0.25 μm apart from the mid superficial dorsal horn at L3 spanning lamina I and II. ***A: ***Neurobiotin (green), after application to all 6 lumbar dorsal roots. ***B: ***VGluT2-IR (magenta). ***C: ***merged image of ***A ***and ***B***, with terminals containing both Neurobiotin and VGluT2-IR appearing whiter. VGluT2-IR terminals labeled with Neurobiotin are abundant in upper lamina I (arrows, and see enlargements of boxed area in ***C***; ***D***-***F***). VGluT2-IR terminals without Neurobiotin also are present in lamina I. A second band of dense VGluT2-IR terminals is visible in outer lamina II, but none are labeled with Neurobiotin in this example. VGluT2-IR varicosities are overall less dense in inner lamina II. Few VGluT2-IR varicosities in inner lamina II contain Neurobiotin: they are mostly large varicosities only weakly labeled with VGluT2-IR (arrowheads; and see enlargements of boxed area in ***C***; ***G***-***I***). Scale bar in ***C ***represents 10 μm and also applies to ***A ***and ***B***. Scale bar in ***I ***represents 5 μm and also applies to ***D***-***H***. LT; Lissauer's Tract, I; lamina I, IIo; outer lamina II, Iii; inner lamina II.

**Figure 4:**
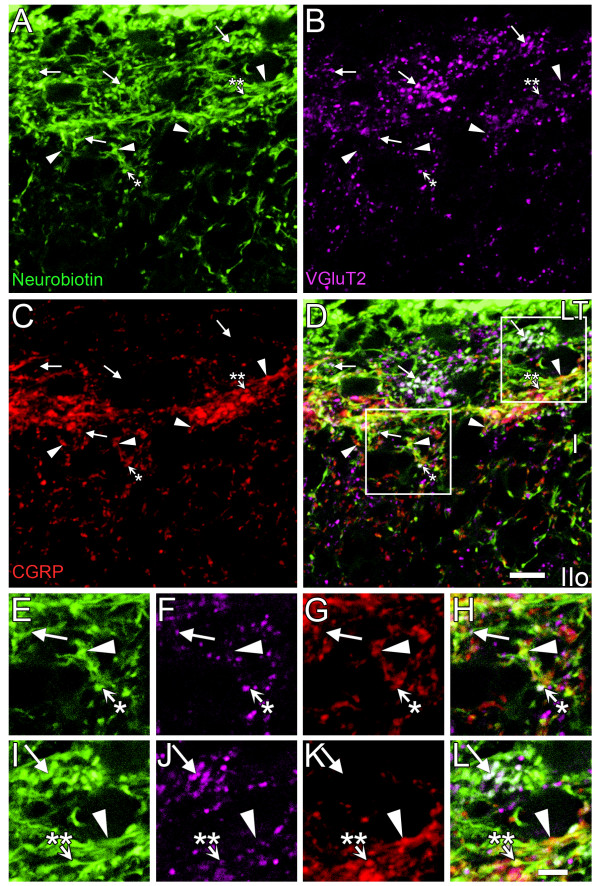
**Expression of VGluT2 in Neurobiotin-labeled primary afferent terminals**. High resolution confocal microscope imaging was performed to identify VGluT2-IR terminals labeled with Neurobiotin and therefore of primary afferent origin. Images represent a projection of 3 optical z-sections taken 0.25 μm apart. ***A: ***Neurobiotin (green), ***B: ***VGluT2-IR (magenta), ***C: ***CGRP-IR (red) and ***D: ***merged image. Varicosities containing both Neurobiotin and VGluT2-IR were common in upper lamina I, and many lacked CGRP-IR (arrows). Conversely, many of the varicosities containing both Neurobiotin and CGRP-IR lacked VGluT2-IR (arrowheads). Occasionally both VGluT2-IR and CGRP-IR occurred within the same Neurobiotin-labeled varicosity (short arrows with asterisks). The arrow with a single asterisk shows an example of a Neurobiotin-labeled varicosity with strong VGluT2-IR and weak CGRP-IR. The arrow with the double asterisks shows an example of a Neurobiotin-labeled varicosity with weak VGluT2-IR and strong CGRP-IR. Varicosities labeled with both Neurobiotin and VGluT2-IR were scarce below lamina I. Panels E-H and I-L are enlargements of regions indicated by white boxes in D. Scale bar represents 10 μm in D (also applies to panels A-C) and 5 μm in L (also applies to panels E-K). LT; Lissauer's Tract, I; lamina I, IIo; outer lamina II, Iii; inner lamina II.

**Figure 5:**
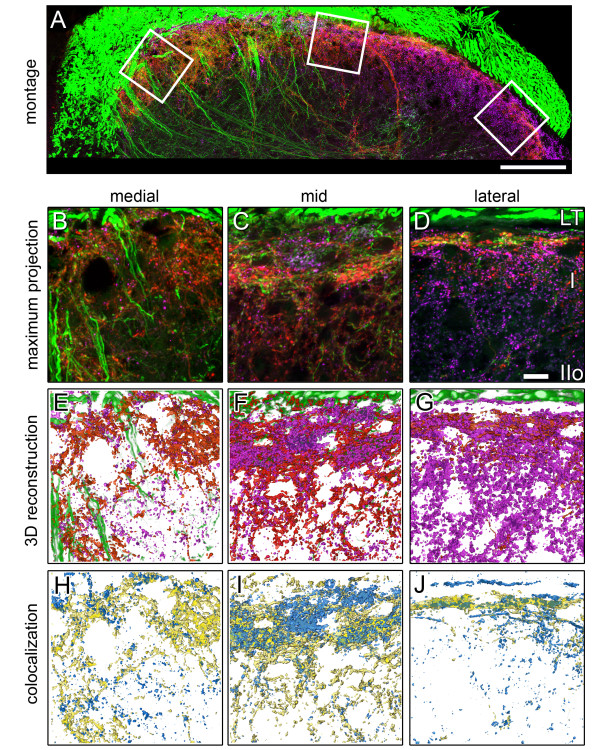
**Identification of VGluT2-IR and CGRP-IR afferents in lamina I using 3D co-localization with Neurobiotin labeling**. ***A: ***Montage of maximum projections of confocal image stacks (11 images per stack; z-interval = 1 μm) showing primary afferents labeled with Neurobiotin (green) and terminals labeled for VGluT2-IR (magenta) and CGRP-IR (red). Representative locations for high resolution analyses of medial, mid and lateral regions are indicated by white boxes. ***B-D: ***maximum projections of high resolution confocal image stacks (41 images per stack; 80 × 80 μm; z-interval = 0.5 μm) from medial (***B***), mid (***C***) and lateral (***D***) regions of lamina I (boxed areas in ***A***). ***E-G: ***3D reconstructions of data from image stacks in ***B-D***, respectively, showing all Neurobiotin-labeled fibers (green), VGluT2-IR terminals (purple) and CGRP-IR terminals (red). ***H-J: ***3D colocalization showing only Neurobiotin-labeled terminals containing VGluT2-IR (blue) or CGRP-IR (yellow). Scale bar represents 100 μm in ***A***. Scale bar in ***D ***represents 10 μm and applies to panels ***B-J***. LT; Lissauer's Tract, I; lamina I, IIo; outer lamina II.

Single optical slice and 3D colocalization analysis of confocal z-stacks spanning lamina I and II (from Lissauer's Tract to the outer border of lamina III) showed that Neurobiotin-labeled VGluT2-IR varicosities occurred predominantly in outer lamina I (Figure 3, see also Figure 4 and Figure 5). Whilst Neurobiotin-labeled VGluT2-IR varicosities were common in upper lamina I, many VGluT2-IR varicosities in lamina I lacked Neurobiotin labeling (Figure 3A-F). Terminals which contained the tracer were scarce in inner lamina I and among the band of VGluT2-IR terminals in outer lamina II. Only occasional VGluT2-IR terminals in inner lamina II contained Neurobiotin. These terminals were mostly of a large size and only weakly labeled for VGluT2-IR compared with Neurobiotin-labeled VGluT2-IR terminals in lamina I (Figure 3A-C, G-I).

VGluT2-IR terminals lacking Neurobiotin labeling presumably included both the terminals of intrinsic spinal neurons as well as VGluT2-IR primary afferent terminals which did not take up Neurobiotin (see below).

### Most VGluT2-IR terminals of primary afferent origin lacked detectable expression of CGRP-IR. Conversely, most CGRP-IR terminals lacked detectable expression of VGluT2-IR

**Figure 6:**
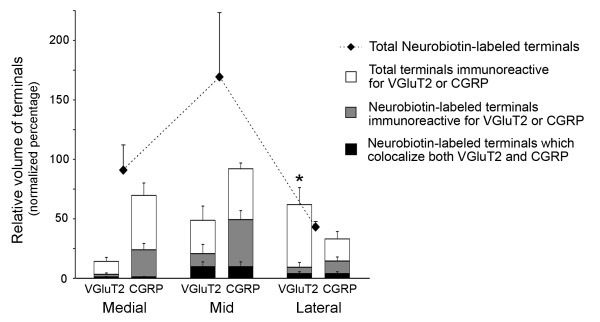
**Distribution of anterogradely-labeled VGluT2-IR and CGRP-IR terminals across lamina I**. Quantification of total and anterogradely-labeled VGluT2 and CGRP immunoreactivity in medial, mid and lateral regions of lamina I. 3D colocalisation analysis and volume quantification was performed on regions of interest spanning lamina I within the high resolution confocal z-stacks at each location as shown in Figure 4. Analysed regions extended from the boundary of Lissauer's Tract and lamina I to a depth of 60 μm within the dorsal horn, which spanned the region where anterogradely-labeled VGluT2-IR terminals were observed. The dimensions of each analysed region was 80 × 60 × 10 μm. Values are expressed as relative volumes normalized against the maximum volume of CGRP-IR terminals in any of the regions for each dorsal horn section. The relative proportion of Neurobiotin-labeled VGluT2-IR terminals compared with the total volume of VGluT2-IR terminals was significantly less in lateral regions of lamina I compared with medial and mid regions of lamina I (asterisk: p ≤ 0.05 for this comparison). The relative proportion of Neurobiotin-labeled CGRP-IR terminals compared with the total volume of CGRP-IR terminals was consistent across all three regions. Two-way ANOVA with Bonferroni post-hoc comparisons; n = 6 sections from 4 animals.

High resolution confocal z-stacks from medial, mid and lateral regions of the outer 60 μm of the dorsal horn (including all of lamina I) were analysed by examination of single optical slices and by using 3D colocalization to compare the spatial distributions of Neurobiotin-labeled VGluT2-IR and Neurobiotin-labeled CGRP-IR terminals (Figure 4, 5, 6). This region included almost all Neurobiotin-labeled VGluT2-IR terminals (Figure 3).

Comparison of Neurobiotin-labeled VGluT2-IR terminals and CGRP-IR terminals by high resolution confocal microscopy of the superficial dorsal horn revealed that the central terminals of these two populations of primary afferents were mostly discrete from one another. Many VGluT2-IR terminals, across a range of VGluT2-IR labeling intensities and varicosity sizes, lacked detectable CGRP expression, as shown by analysis of individual optical sections (Figure 4) and by comprehensive 3D colocalisation analysis of confocal z-stacks (Figure 5, 6). Similarly most CGRP-IR terminals lacked detectable VGluT2-IR expression (Figure 4, 5, 6). Nevertheless, a small proportion of Neurobiotin-labeled primary afferent terminals did express both VGluT2-IR and CGRP-IR. Some Neurobiotin-labeled terminals with strong VGluT2-IR labeling showed only weak CGRP-IR labeling, and vice versa. In some cases, different intensities of VGluT2-IR and CGRP-IR occurred within the same Neurobiotin-labeled varicosity, as has been previously reported [27].

Many, but not all, CGRP-IR terminals in lamina I at the L3 spinal level were labeled with Neurobiotin. Across the medial, mid and lateral regions spanning lamina I which were analysed for the six experiments, 44 ± 4% of CGRP-IR terminals were found to contain the tracer (Table 1).

**Table 1:** Neurobiotin labeling of VGluT2-IR and CGRP-IR terminals in lamina I

	Medial	Mid	Lateral	Overall
Neurobiotin-labeled terminals with CGRP-IR as proportion of volume of total CGRP-IR terminals	37 ± 10%	53 ± 7%	42 ± 6%	44 ± 4%

Neurobiotin-labeled terminals with VGluT2-IR as proportion of volume of total VGluT2-IR terminals	26 ± 10%	39 ± 10%	13 ± 4%*	26 ± 4%

Neurobiotin-labeled VGluT2-IR terminals as proportion of Neurobiotin-labeled CGRP-IR terminals	69 ± 12%	70 ± 9%	28 ± 7%*	56 ± 4%

The proportion of VGluT2-IR terminals which were labeled with Neurobiotin as well as the ratio of Neurobiotin-labeled VGluT2-IR terminals compared to Neurobiotin-labeled CGRP terminals were significantly lower in lateral lamina I compared to medial and mid lamina I (two-way ANOVA with Bonferroni corrections using 95% confidence limits; p < 0.05, indicated by asterisks). Analysis of L3 spinal cord sections, n = 6 animals.

Since all CGRP-IR terminals are believed to be of primary afferent origin, the presence of CGRP-IR terminals without Neurobiotin label indicated that not all afferent fibers were labeled with this procedure. Primary afferent terminals within the L3 dorsal horn which originate from DRG at other, untraced, segmental levels would also remain unfilled by Neurobiotin.

### Quantitative 3D analysis of the non uniform projections of VGluT2-IR and CGRP-IR primary afferents to lamina I

Quantitative 3D analysis of confocal data sets confirmed our wide-field observations. Averaged across lamina I, total CGRP-IR terminals were more abundant than total VGluT2-IR terminals (CGRP-IR terminals: 65.3 ± 7.2% normalized volume; VGluT2-IR terminals: 41.9 ± 7.7% normalized volume). Nevertheless, the highest density of total VGluT2-IR terminals occurred in lateral lamina I, where they outnumbered total CGRP-IR terminals (lateral region VGluT2-IR terminals: 62.2 ± 14.4% normalized volume; lateral region CGRP-IR terminals: 33.3 ± 6.2% normalized volume; see Figure 6). In contrast, the highest density of CGRP-IR terminals occurred in mid lamina I.

The overall distribution of Neurobiotin-labeled fibers in lamina I followed that of CGRP-IR terminals, with highest density in the mid region. Neurobiotin-labeled CGRP-IR terminals were more abundant than Neurobiotin-labeled VGluT2-IR terminals in each of the three lamina I regions (Figure 6).

The proportion of CGRP-IR terminals labeled with Neurobiotin was comparable across all three regions of lamina I (44 ± 4%; Figure 6, Table 1). However, the proportion of VGluT2-IR terminals labeled with Neurobiotin was significantly lower in the lateral region compared to mid and medial regions of lamina I (two-way ANOVA with Bonferroni corrections; p ≤ 0.05), indicating that lateral lamina I contained predominantly intrinsic VGluT2-IR terminals (Figure 6, Table 1). This interpretation was confirmed by comparing the Neurobiotin labeling efficiency of CGRP-IR and VGluT2-IR terminals across lamina I. In medial and mid regions, the proportion of VGluT2-IR terminals labeled with Neurobiotin compared with the proportion of CGRP-IR terminals labeled with Neurobiotin was 70 ± 6%, whilst in lateral regions, this ratio was only 28 ± 7% (Table 1).

### Few primary afferents contain both VGluT2-IR and CGRP-IR

Throughout lamina I, less than half (38 ± 4%) of Neurobiotin-labeled VGluT2-IR terminals co-expressed CGRP-IR (Table 2, Figure 6). Less than 20% of all Neurobiotin-labeled CGRP-IR terminals contained detectable VGluT2-IR (Table 2, Figure 6). The highest density of Neurobiotin-labeled terminals colocalizing both VGluT2-IR and CGRP-IR occurred in the mid region of lamina I (10.0 ± 4.0% of Neurobiotin-labeled terminals; Figure 6).

**Table 2:** Colocalization of VGluT2-IR and CGRP-IR in Neurobiotin-labeled terminals across lamina I

	Medial	Mid	Lateral	Overall
Neurobiotin-labeled VGluT2-IR terminals with CGRP-IR^†^	39 ± 7%	44 ± 8%	32 ± 8%	38 ± 4%

Neurobiotin-labeled CGRP-IR terminals with VGluT2-IR^‡^	7 ± 2%	20 ± 7%	24 ± 10%	17 ± 4%

^†^Neurobiotin-labeled VGluT2-IR terminals with CGRP-IR as a proportion of total Neurobiotin-labeled VGluT2 terminals in lamina I by volume.

^‡^Neurobiotin-labeled CGRP-IR terminals with VGluT2-IR as a proportion of total Neurobiotin-labeled CGRP terminals in lamina I by volume.

Analysis of L3 spinal cord, n = 6 animals.

### Clustered arrangement of VGluT2-IR and CGRP-IR terminals in lamina I

**Figure 7:**
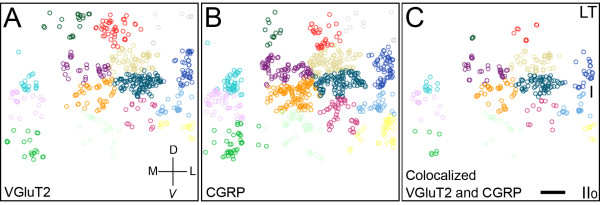
**Cluster analysis of anterogradely-labeled VGluT2-IR and CGRP-IR terminals in lamina I**. The 3D center of mass coordinates were calculated for the Neurobiotin- and immuno-labeled terminals from 3D confocal data sets for medial, mid and lateral lamina I from one animal. The coordinates were analysed by hierarchical cluster analysis with SPSS. The data set shown here is from medial lamina I. ***A: ***Neurobiotin-labeled terminals with VGluT2-IR; ***B: ***Neurobiotin-labeled terminals with CGRP-IR; ***C: ***Neurobiotin-labeled terminals with both VGluT2-IR and CGRP-IR. Each statistically-determined cluster is assigned a different color. The red and dark green clusters at the dorsal edge of the field are dominated by VGluT2-IR terminals (ratio of VGluT2:CGRP terminals is about 2:1 in each cluster), whilst the orange and yellow clusters along the ventral edge of the field are dominated by CGRP-IR terminals (ratio of CGRP:VGluT2 terminals is about 2:1 in each cluster; *X^2 ^*test and analysis of residuals, p < 0.05 in each case). The axes indicate the orientation of the data set: D; dorsal, V; ventral, M; medial, L; lateral. Scale bar in C represents 10 μm and applies to panels ***A-C***. LT; Lissauer's Tract, I; lamina I, IIo; outer lamina II.

Analysis of 3D confocal data sets confirmed wide-field image analyses indicating that VGluT2-IR terminals and CGRP-IR terminals commonly formed clusters within lamina I. Such clusters of terminals appeared to surround or abut spaces that contained unlabeled nerve cell bodies (Figure 5B-G). The respective subsets of VGluT2-IR and CGRP-IR terminals which were Neurobiotin labeled also formed clusters surrounded spaces that contained unlabeled nerve cell bodies (Figure 5H-J). Cluster analysis of the 3D center of mass coordinates of 3899 Neurobiotin-labeled terminals across lamina I showed that VGluT2-IR terminals and CGRP-IR terminals tended to be clustered together more than would be expected for a random distribution (Figure 7). All clusters detected this way contained both terminals which were VGluT2-IR and terminals which were CGRP-IR. However, 35% of clusters were significantly enriched in either VGluT2-IR or CGRP-IR terminals (*X*^2 ^tests and analysis of residuals, p < 0.05; hierarchical cluster analysis using 15 random seed points). Most clusters also contained a small number of Neurobiotin-labeled terminals that co-expressed both VGluT2-IR and CGRP-IR (Figure 7C).

## Discussion

### VGluT2-IR primary afferents project to lamina I

We have positively identified and mapped the distribution of the central terminals of non-peptidergic VGluT2-expressing primary afferents in mouse lumbar spinal cord. These terminals were mostly restricted to lamina I, and only occasionally observed in lamina II, where IB4-binding small-diameter afferent terminals project. Therefore we also compared the distribution of VGluT2-expressing primary afferents with that of peptidergic primary afferents in lamina I. Previous studies have shown that VGluT2-IR labels a subpopulation of cell bodies within DRG, including neurons of both small (cross sectional area < 300 μm^2^) and medium (cross sectional area 300-600 μm^2^) soma size [26,30]. Only some of these VGluT2-IR cell bodies, whether small or medium-sized, co-express CGRP [26,30]. Based on the predominant projection of fine diameter VGluT2-expressing primary afferents into lamina I, it is most probable that the non-peptidergic VGluT2-IR neurons include a class of unmyelinated or lightly myelinated nociceptors [27]. These non-peptidergic VGluT2-IR primary afferent terminals also may include non-nociceptive Aδ and C-fibers such as innocuous thermal and mechanical afferents.

The central terminals of these putative glutamatergic nociceptors were largely restricted to the outer 60 μm of lamina I. Their medial-to-lateral distribution across lamina I was not different overall from the projection of CGRP-IR afferents into lamina I. In contrast, the total density of all VGluT2-IR terminals was much higher in the lateral regions of lamina I. Most lateral VGluT2-IR terminals were not labeled by anterograde tracing from the dorsal roots and therefore they are most likely derived from central neurons. The high ratio of intrinsic to primary afferent VGluT2-IR terminals in the dorsal horn is consistent with observations showing minimal depletion of VGluT2-IR from the dorsal horn following dorsal rhizotomy in rats [25,28,29] or selective knock-out of VGluT2 expression in nociceptors of mice [26].

Several previous studies have reported that VGluTs are either undetectable or present at very low levels in most small diameter peptidergic afferents and their central projections, in both mice [28,32] and rats [19,24,25,27-29,31,37]. Similarly, immunoreactivity to the amino acids themselves is low or undetectable in the soma of many peptidergic afferents [6]. Nevertheless, more recent reports find that almost all mouse DRG neurons which express CGRP-IR or bind IB4 do also express VGluT2 at some level and are functionally glutamatergic [26,38]. Regardless of these apparent differences, all studies have illustrated significant VGluT2 expression in a population of small-medium DRG neurons which lack CGRP and IB4-binding, whilst finding only low or undetectable levels of VGluT2-IR in the central terminals of many peptidergic primary afferents. Our data have confirmed that the central terminals of anterogradely-labeled VGluT2-expressing terminals in C57/Bl6 mice are mostly distinct from IB4-binding terminals, and that only a small proportion of anterogradely-labeled afferent terminals in lamina I contain both CGRP-IR and detectable VGluT2-IR. Nevertheless, around 17% of CGRP-IR terminals in the dorsal horn also contained detectable VGluT2-IR. Therefore, this combination of labels identifies three potential neurochemical phenotypes within small diameter afferents projecting to lamina I: those with CGRP-IR, those with VGluT2-IR, and those with both (cf rat DRG: [6]). Most CGRP-IR projections in lamina I would be expected also to contain substance P [30]. No other peptides are known to be expressed by the VGluT2-IR afferents that lack CGRP-IR.

Recently, several populations of small-diameter primary afferents have been described that neither express neuropeptides nor bind IB4, including most transient receptor potential cation channel subfamily M (TRPM8)-expressing nociceptors [39,40], some TRPV1-expressing nociceptors [41], and small subpopulations of Mrgprd-expressing and TRP Subfamily A (TRPA1)-expressing nociceptors [42]. Given the recent demonstration of widespread expression of VGluT2 in small diameter afferents [26,38], we predict that the non-peptidergic VGluT2-IR neurons we have identified must include at least some of these populations. Taken together, these studies demonstrate that the commonly used binary classification of small diameter nociceptors as either peptide-expressing or non-peptidergic IB4 lectin-binding neurons is insufficient to accommodate the high degree of heterogeneity of small-diameter afferents (for further critique of nociceptor classification, see [17]).

There is considerable functional evidence that glutamate mediates fast synaptic transmission from the great majority of primary afferents. In contrast, neuropeptide transmitters such as substance P and CGRP are thought to have mainly a modulatory role, enhancing the excitability of the second order neurons, and therefore require the co-release of an excitatory amino acid in order to excite second order neurons in the dorsal horn. Indeed a recent study in mice in which the VGluT2 gene was selectively ablated in the majority of nociceptors demonstrated that responses to acute noxious heat, mechanical and chemical stimuli, as well as heat hyperalgesia induced by injury or neuropathic pain, are dependent on VGluT2-dependent glutamate transmission [26]. Whilst the usual interpretation is that peptides and glutamate may be co-released from the same afferent neuron, functional studies to date do not preclude the possibility that peptide and amino acid release may occur simultaneously from different afferent neurons which converge on lamina I neurons. There is pharmacological evidence consistent with the independent release of glutamate or neuropeptides, presumably from different subpopulations of nociceptive neurons [43,44]. Thus it is possible that peptide release from nociceptors that may lack significant VGluT2 expression may be involved in modulation of responses to acute noxious heat, mechanical and chemical stimuli and heat hyperalgesia induced by injury or neuropathic pain. Indeed, peptide release from nociceptors lacking VGluT2-expression may also play a role in the so-called "VGluT2-independent" processes identified in [26], such as injury induced mechanical allodynia.

Whilst several reports have identified that some peptidergic sensory neurons lack VGluT2-IR in their somata [26,30] and their terminals [27-31,37,45] there is considerable variation regarding the proportion of peptidergic neurons this population represents. Whilst different antibodies were used by Scherrer *et al*. 2010 [26] and the present study, the distribution of VGluT2-IR terminals in the spinal dorsal horn is remarkably similar in both studies. It is possible that different strains of mice have different compositions of nociceptor populations, as different mouse strains can exhibit considerable differences in their response to different types of noxious stimuli [46]. A systematic study of the colocalisation of the common nociceptor markers including VGluT2, CGRP, IB4 and TRPV1 would be required across the common laboratory mouse strains to examine any relationship between nociceptor subpopulations and their phenotypes.

### Convergence of primary afferents in lamina I

We have found that putative non-peptidergic glutamatergic and peptidergic primary afferent terminals cluster into partially overlapping domains within lamina I. This arrangement suggests that separate populations of nociceptors converge onto second order neurons in lamina I. Indeed, a recent analysis of VGluT2-IR and CGRP-IR boutons contacting lamina I projection neurons in rat spinal cord found that many CGRP-IR boutons either lacked VGluT2-IR or were weakly immunoreactive for VGluT2 [37]. Pharmacological studies showing dissociated release of glutamate and peptides in the dorsal horn [43,44] are consistent with these immunohistochemical observations. Electrophysiological evidence that many lamina I neurons receive convergent C-fiber and Aδ-fiber nociceptive inputs from different dorsal root levels [35] implies yet another level of processing of diverse inputs by lamina I neurons.

Our immunohistochemical methods do not allow the direct detection of synaptic inputs from different classes of primary afferents to lamina I neurons. However, at least some of the Neurobiotin-labeled VGluT2-IR terminals must be responsible for fast glutamatergic synaptic transmission to lamina I neurons. Peptidergic afferents could communicate with lamina I neurons by non-synaptic transmission of neuropeptides (volume transmission) as proposed many years ago [47]. If so, spatial clustering of peptidergic boutons need not necessarily limit the actions of peptides released from primary afferents to their nearest lamina I neurons.

Most fast transmission from nociceptors to lamina I neurons appears to be mediated by glutamate [48]. Peptidergic transmission is mediated mainly by substance P whose actions are facilitated by CGRP [49-51]. Various genetic and pharmacological manipulations of substance P-mediated transmission into the dorsal horn suggest that these peptides mediate only a subset of noxious sensations [48,52,53]. Indeed, conditional knock-out of VGluT2 expression in small diameter neurons indicates that peptides alone probably cannot sustain nociceptive transmission in the dorsal horn [26].

VGluT2-IR terminals have been found in the skin [32]. However, the peripheral projections of non-peptidergic VGluT2-IR afferents are not known in detail. Nevertheless, the co-distribution of their central terminals together with CGRP-IR terminals across the dorsal horn implies that each population of afferents has a similar somatotopic organization reflecting comparable peripheral projections [54]. Therefore, when glutamate and peptides such as substance P and CGRP all contribute to nociceptive responses of lamina I neurons, our observations suggest two possible pathways: either (1) all this transmission is mediated by the small proportion of primary afferents co-expressing peptides and high levels of VGluT2; or (2) separate populations of non-peptidergic glutamatergic and peptidergic nociceptors are co-activated and converge onto common targets in lamina I. Even if the central terminals of peptidergic afferents do contribute to glutamatergic nociceptive transmission, perhaps either underpinned by undetectable levels of VGluT2, as recently suggested [26], or through a novel exocytosis mechanism [55], our data clearly demonstrate a previously unrecognized level of organization in lamina I of the dorsal horn consistent with the presence of multiple nociceptive processing pathways.

## Methods

### Anterograde axonal tracing of primary afferent central terminals

C57/Bl6 mice (8-10 weeks old) were deeply anaesthetized with inhaled isoflurane (Abbott Australasia Pty Ltd, NSW, Australia) then killed by heart removal. The lumbar spinal cord and attached dorsal roots were dissected under oxygenated HEPES buffered balanced salt solution (146 mM NaCl, 4.7 mM KCl, 0.6 mM MgSO_4_, 1.6 mM NaHCO_3_, 0.13 mM NaH_2_PO_4_, 7.8 mM D-glucose, 2.5 mM CaCl_2_, 0.1 mM ascorbic acid, 20 mM HEPES, pH 7.3). Spinal cord segments with attached dorsal roots were either prepared for anterograde axonal tracing with Neurobiotin *in vitro *or fixed immediately and processed for immunohistochemistry. Experiments were approved by the Animal Welfare Committee of Flinders University.

For anterograde axonal tracing, the distal ends of dorsal roots (single L3 dorsal root, unilateral; all six lumbar dorsal roots (L1-L6) unilateral plus L3 dorsal root contralateral; or L1-L6 dorsal roots, unilateral) were placed within internal compartments of a dissecting culture dish with the attached spinal cord in the outer compartment. The exchange of solutions between compartments was blocked with petroleum jelly and coverglass barriers. To aid uptake of the tracer, the distal end of the dorsal roots were washed three times in Ca^2+ ^free artificial intracellular solution (AIS; 7 mM MgCl_2_, 5 mM D-glucose, 1 mM EGTA, 5 mM ATP, 20 mM HEPES, 0.02% saponin, 1% dimethyl sulfoxide (DMSO), 100 IU/ml penicillin, 100 μg/ml streptomycin, 10 μg/ml gentamicin) and a fresh cut of the distal end(s) performed during the first AIS wash. 10-20 μl of 5% Neurobiotin (*N*-(2-aminoethyl) biotinamide hydrochloride; Vector Laboratories, Burlingame, CA) in AIS [56] was applied to the dorsal roots and covered with paraffin oil. The HEPES buffered balanced salt solution in the outer compartment was replaced with Dulbecco's modified Eagle Medium F12 (DMEM/F12) supplemented with 10% heat-inactivated fetal calf serum, 14.3 mM NaHCO_3_, 2.8 mM CaCl_2_, 100 IU/ml penicillin and 100 μg/ml streptomycin and the preparation incubated in a humidified cell culture incubator with 5% CO_2 _at 37°C for 4 hours. The spinal cord and attached dorsal roots were then fixed and processed as described below.

### Optimisation and assessment of anterograde axonal tracing using Neurobiotin

Anterograde axonal tracing with Neurobiotin *in vitro *was originally developed to identify peripheral projections of sensory neurons in the gastrointestinal tract [56]. As this study represents the first reported use of Neurobiotin to bulk label primary afferent terminals in mouse spinal cord by anterograde axonal tracing from the dorsal roots, the technique was carefully optimized and validated. The optimal incubation time for transport of Neurobiotin into the spinal dorsal horn was determined in preliminary experiments. Consequently, 4 hours incubation time was used for definitive experiments as at least 2.5 hours incubation was required to achieve maximal tracing of fine diameter primary afferents in the dorsal horn, but incubation for more than 6 hours commonly resulted in deterioration of the tissue with no further increase in the extent of labeling. Comparable labeling of primary afferent terminals was seen with 5% or 1% Neurobiotin. Minor variation in the extent of tracing of fibers in the dorsal horn was observed between experiments, presumably due to low level accidental damage to the nerve roots during the dissection procedure. The six experiments chosen for data analysis all showed comprehensive Neurobiotin transport extending into superficial and deep dorsal horn laminae across several spinal segments, extending into the ventral horn at L3. On average, 44.4 ± 4% of CGRP-IR fibers in the superficial dorsal horn were labeled with Neurobiotin in these six experiments (Table 1). As all CGRP-IR terminals in the superficial dorsal horn are of primary afferent origin, this value is an indicator of the anterograde tracing efficiency of primary afferent fibers. There was no evidence of preferential tracing of CGRP-IR primary afferents compared to VGluT2-IR primary afferents (see Results).

### Tissue processing and immunohistochemistry

Spinal cord segments were fixed for 24-72 hours in Zamboni's fixative (2% formaldehyde and 0.5% picric acid in 0.1 M phosphate buffer, pH 7.0), dehydrated through a graded series of ethanol, cleared in DMSO and embedded in polyethylene glycol (MW1450) [57]. Serial transverse 20 μm sections were cut on a rotary microtome and stored in phosphate-buffered saline (PBS, pH 7.0) with 0.01% sodium azide. Neurobiotin-labeled terminals were detected using streptavidin conjugated to dichlorotriazinylamino fluorescein (DTAF; Jackson ImmunoResearch, West Grove, PA). Immunofluorescence was done as previously described using antibodies to VGluT2 and CGRP which have already been well characterized in mouse spinal cord (see below). Sections were preincubated in 10% normal donkey serum (NDS) for 30 minutes before incubation for 48-72 hours in primary antisera containing 10% NDS. Primary antisera were washed off in PBS and the sections incubated overnight in species-specific secondary antisera raised in donkeys and conjugated to Cy3 and Cy5 respectively (Jackson ImmunoResearch). In some sections, the isolectin IB4 (from *Griffonia simplicifolia*) conjugated to AlexaFluor488 (code I-21411, Molecular Probes, Eugene, OR) was included with the primary antisera, Neurobiotin was detected with streptavidin conjugated to Cy5 (Jackson ImmunoResearch) and goat anti-CGRP was detected with donkey anti-goat IgG conjugated to aminomethylcoumarin acetate (AMCA; Jackson ImmunoResearch). Serial sections were mounted in 0.5 M sodium carbonate-buffered glycerol (pH 8.6) and coverslips were sealed with nail varnish.

### Antibody Characterisation

The primary antibodies used in this study have already been well characterized for mouse spinal cord. The VGluT2 polyclonal antibody was raised in rabbit against a fusion protein containing glutathione *S*-transferase (GST) and amino acid residues 510-582 of rat VGluT2 (catalog number 135 102, Synaptic Systems, Göttingen, Germany). We have previously demonstrated that labeling with this same batch of antibody in mouse spinal cord was completely abolished by prior incubation with the control blocking protein, 135-1P (Synaptic Systems, Göttingen, Germany) [30]. Earlier and subsequent batches of this polyclonal antibody have been shown to detect a 65 kD protein in rat brain and spinal cord extracts in Western blot, with labeling completely blocked by pre-incubation with the antigen used for the immunisation [58,59]. Immunolabeling of spinal cord with this VGluT2 antibody was consistent with other reported VGluT2 antibodies [24,25,27,29,32] and labeling of VGluT2 with the same antibody in rat spinal cord [28].

The CGRP polyclonal antibody (code number 1780, Arnel Products Co Inc, New York NY) was raised in goat against rat CGRP conjugated to gamma globulin. This antibody has been previously shown not to cross-react with any known unrelated peptides in sensory and autonomic ganglia [60] and exhibits labeling consistent with other reported CGRP antibodies used in guinea-pig, rat and mouse spinal cord [7,28,30].

### Microscopy and image analysis

All images shown and data analyses are from L3 spinal cord unless otherwise specified. For wide field fluorescence microscopy, sections were examined with an Olympus AX70 epifluorescence microscope (Olympus Optical Co. Ltd., Tokyo, Japan) fitted with highly discriminating filters (Chroma Optical, Brattleboro, VT). Images were collected using a Hamamatsu Orca cooled CCD camera (Hamamatsu Photonics, Hamamatsu City, Japan) connected to a PC running AnalySIS FIVE software (v5.0, Olympus Soft Imaging System GmbH, Münster, Germany).

Identification of regions in the spinal dorsal horn were identified visually as follows: Lissauer's Tract; dense band of axons dorsal to spinal cord, Lamina I, region of dense CGRP-IR terminals; lamina II; immediately deep to the predominant band of CGPR-IR in lamina I and containing a band of IB4-binding terminals (outer lamina II) and a secondary band of VGluT2-IR terminals (inner lamina II), lateral spinal nucleus; lateral to the lateral boundary of the dorsal horn (marked by lateral border of CGRP-IR), the lateral spinal nucleus is devoid of CGRP-IR and labels intensely for VGluT2-IR.

ImageJ software (Rasband, 1997-2009) was used to generate Fast Fourier Transformations (FFT) of images to create a band-pass spatial filter selectively preserving low-frequency data representing the dominant spatial features of labeled terminals in lamina I. The FFT images were manually edited to produce a low-frequency band pass filter and reverse transformed. 3D intensity plots of FFT-filtered data were prepared in ImageJ, from merged images of sections from 3 animals manually adjusted to correct for variation in dorsal horn dimensions. Additionally, the spatial coordinates and sizes of domains derived from FFT-filtered data from 5 consecutive sections from one animal were analysed statistically after dividing the dorsal horn into medial and lateral regions. Domain coordinates (center of mass) and size (area) were obtained from the FFT images in ImageJ, and analysed in SPSS v17 (SPSS Inc, Chicago, Illinois).

Confocal imaging was done with a Leica TCS SP5 laser scanning confocal microscope and Leica Application Suite Advanced Fluorescence software (Leica Microsystems GmbH, Wetzlar, Germany) in sequential scanning mode to avoid any chance of signal bleed-through between channels. Confocal z-stacks, consisting of 41 optical sections, 0.25 μm apart (total depth of 10 μm), were taken with a 63 × 1.4 N.A. oil immersion objective and 3× digital zoom with resultant pixel size of 80 nm. Z-stacks of medial, mid and lateral dorsal horn were aligned parallel with Lissauer's tract and the dorsal-most part of lamina I. At each location, two further z-stacks were acquired 75 μm and 150 μm ventral to the lamina I stacks, thereby generating a montage of three z-stacks spanning from Lissauer's tract into lamina III. The z-stacks were analysed with Avizo Fire software (v5.1.0, Mercury Computer Systems Inc, Merignac, France) for 3D reconstruction, 3D quantification and 3D colocalization.

For 3D analysis, data sets from each detection channel were 3D-median filtered (kernel size 3) to reduce single pixel noise, thresholds were set to select specific labeling of nerve terminals above background labeling and the "exclusive AND" operator was used to determine colocalization between the three fluorescence data channels in x-y-z coordinates. All thresholds were set according to structural criteria visualising the nerve fibres and their terminals in 3D and all thresholds were confirmed to be at least two standard deviations above the mean intensity value of the background for each respective fluorescence data channel. Identical thresholds were used for each of the analysed regions within a spinal cord section and the respective thresholds for each channel were verified to be consistent between sections and between experiments. For each z-stack, data sets representing the anterogradely-labeled subset of VGluT2-IR terminals (Neurobiotin and VGluT2-IR colocalized in 3D), the anterogradely-labeled subset of CGRP-IR terminals (Neurobiotin and CGRP-IR colocalized in 3D) and the anterogradely-labeled subset of terminals colocalizing both VGluT2-IR and CGRP-IR (Neurobiotin, VGluT2-IR and CGRP all colocalized in 3D) were calculated from the three original data channels and designated as new data sets. For illustration purposes, Neurobiotin-labeled primary afferents are shown as volume rendered data sets, while subsets of VGluT2-IR and CGRP-IR terminals (total immunoreactivity and those anterogradely labeled with Neurobiotin) are displayed with isosurface rendering. Since anterogradely-labeled VGluT2-IR terminals were scarce below the outer 60 μm of lamina I, quantification was done on a region of interest (ROI) which extended from the boundary of Lissauer's Tract and lamina I to a depth of 60 μm within lamina I (ROI: 80 μm × 60 μm × 10 μm). The volume of each 3D data set (total anterogradely-labeled fibers; total and anterogradely-labeled subsets of VGluT2-IR and CGRP-IR terminals; and the total and anterogradely-labeled terminals colocalizing both VGluT2-IR and CGRP-IR) was calculated and exported into Microsoft Office Excel (Microsoft Coorporation, US) for further analysis. For comparisons across experiments, volume data were normalized to the maximum volume of CGRP-IR structures in any of the three ROIs for each dorsal horn section. Data are displayed as the mean ± standard error of the mean. Statistical analyses were done with SPSS.

For analysis of the spatial arrangement of VGluT2-IR and CGRP-IR terminals, z-stacks were processed in Avizo Fire software. Data which had been 3D median-filtered, thresholded and analysed for 3D colocalization as described above were further processed by an "Opening" operand to enable separation of adjoining terminals into discrete entities. The x-y-z coordinates of the center of mass of each derived structure, along with its corresponding volume, were computed in Avizo Fire and their spatial distributions were analysed in SPSS with hierarchical cluster analysis, using 10-20 cluster seeds. The cluster analyses used both weighted coordinates (adjusted by volume for the different number of terminals represented in each 3D structure) and unweighted coordinates in 3D (x-y-z coordinates) and in 2D (x-y coordinates). Each cluster was then analysed to determine if it contained more CGRP-IR or VGluT2-IR terminals than would be expected by chance using *X^2 ^*statistics and analysis of residuals. One complete data set from each dorsal horn location (medial, mid and lateral regions of lamina I) was analysed in this way.

## List of abbreviations

3D: three-dimensional; AIS: artificial intracellular solution; CGRP: calcitonin gene-related peptide; FFT: fast fourier transformation; IB4: isolectin B4; IR: immunoreactive; ROI: region of interest; TRP: transient receptor potential cation channel; VGluT: vesicular glutamate transporter.

## Competing interests

The authors declare that they have no competing interests.

## Authors' contributions

JNC performed the experiments, participated in the design and analysis, drafted the manuscript and prepared some of the figures. RLA participated in the design of the study, some of the experiments and in preparation of some of the figures. RVH critically revised the manuscript. ILG conceived of the study, oversaw the design and coordination of the study, performed the statistical analyses and critically revised the manuscript. All authors have read and approved the final manuscript.
